# The ethical and regulatory framework of medical artificial intelligence (AI) system’s behavioral performance for image-based diagnostics: An ethical evaluation of the International and European Union Scope

**DOI:** 10.1371/journal.pdig.0001781

**Published:** 2026-10-14

**Authors:** Nina Parchmann, Marcin Orzechowski, Daniel Braun, Florian Steger

**Affiliations:** 1 Institute of the History, Philosophy and Ethics of Medicine, Ulm University, Ulm, Germany; 2 Institute of Neural Information Processing, Ulm University, Ulm, Germany; University of Pittsburgh School of Medicine, UNITED STATES OF AMERICA

## Abstract

The use of Artificial Intelligence (AI) for image-based diagnostics is rising rapidly and enables new opportunities for improving clinical diagnostics through high precision and a quick identification of anomalies. However, a technology characterized by a high variability of image data and the opaqueness of AI models within patient care raises ethical challenges and requires protection by an appropriate ethical and regulatory framework. To identify ethical challenges of the ethical and regulatory framework of the behavioral performance of medical AI systems for image-based diagnostics on the international and European Union level, we searched and screened documents in April and May 2025. The documents were thematically evaluated before conducting an ethical analysis. We identified regulations and ethical recommendations that could be divided into five thematic categories: The (i) data basis is regulated by data standardization, data assessment, and data quality. (ii) Reliability considers assessments, the intended performance, robustness, accuracy, and compliance, whereas the (iii) comprehensibility centers on explainability, understandability, and traceability. The (iv) disclosure of the system considers transparency, operational disclosure, and collaborations, and the (v) usability is regulated on resources and competency. This framework could support the autonomy, beneficence, non-maleficence, and (social) justice, however allows a wide range of compliant technical measures that vary in effectiveness. The insufficient technical feasibility to provide security measures against malicious data alteration as well as the dynamic development of attack and defense mechanisms severely challenge the maintenance of a compliant safe and secure diagnostic AI system. Thereby, the autonomy, beneficence, non-maleficence, and (social) justice could be jeopardized. In conclusion, AI image-based diagnostic systems are regulated by a similar ethical and regulatory framework as general medical AI systems. To protect data and the intended performance, sufficient protection through a regulatory framework is necessary that responds to the challenges of image-based AI diagnostic systems.

## Author summary

The use of Artificial Intelligence (AI) in diagnostics based on medical images is increasing. AI can automatically analyze images, quickly detect patterns, and support physicians. However, using this technology in patient care raises ethical challenges and requires ethical and regulatory protection. In April and May 2025, we screened official documents to identify ethical challenges of the ethical and regulatory framework of the diagnostic technology’s behavior on the international and European Union level. The documents were thematically and ethically analyzed by using the ethical principles of autonomy, beneficence, non-maleficence, and (social) justice. We found that the framework consists of five broad themes: (i) data basis, (ii) reliability, (iii) comprehensibility, (iv) disclosure, and (v) usability. All ethical principles are supported, but they are limited by a wide range of technical methods. Insufficient security measures for protecting data, and the race between developing attack and defense mechanisms, hinder maintaining safety. Thereby, all ethical principles could be harmed. In conclusion, we found that image-based diagnostic AI systems are protected by similar ethical guidance and regulations as medical AI systems for general purposes. We recommend establishing a regulatory framework that addresses the system’s specific challenges in order to sufficiently protect data and its target functions.

## Introduction

The use of Artificial Intelligence (AI) for image-based diagnostics reacts to the growing shortage of radiologists [1,2]. A rapid AI-based analysis of medical images allows a significant saving of time and human resources to provide medical care for the increasing demand of diagnostic imaging [3]. The high performance and precision of AI-based systems enable new opportunities to improve the quality of clinical diagnostics through an early detection of anomalies and a quick identification of patterns in medical images [4]. Due to a large variability of medical image data [5] and the opaqueness of deep learning models [6,7], the development and use of AI-based diagnostic systems pose major challenges. The interdisciplinary research training group KEMAI (Knowledge Infusion and Extraction for Explainable Medical AI) aims for combining knowledge- and learning-based systems to reach state-of-the-art accuracy in image-based diagnosis, while providing clearly communicated results. To ensure a safe, value-based, and intended system performance, establishing an accountable ethical and regulatory framework is essential for building trust, harmonizing interests, and minimizing risks [8].

However, providing an appropriate regulatory framework for a rapidly developing high-risk medical technology raises ethical questions. Therefore, we aimed to identify ethical challenges, chances, and limitations of the ethical and regulatory framework for the behavioral performance of AI systems for image-based diagnostics by examining documents and conducting an ethical analysis. Here, our research focus centered on international and European Union’s (EU) regulations and ethical recommendations.

## Materials and methods

To identify the ethical and regulatory framework of medical AI systems for image-based diagnostics, we first collected data by searching and screening official documents [9,10] that we combined with citation tracking [11] in April and May 2025. This approach allowed a structured search that is broadened by context-specific documents irrespective of a preassigned search string.

To narrow the scope of regulations and to specify the term “behavioral performance” within the diversity of field-specific technical understandings [12], we determined the characteristics of the medical system and its performance: 1) The diagnostic image-based AI system is defined as high-risk AI that is based on machine and deep learning and uses pseudonymized data. 2) The behavioral performance of the medical AI system is defined as a behavior that is composed of prerequisites and characteristics that enable predefined aims and purposes through human and technical components.

The inclusion of documents in the research was based on defined criteria: 1) Documents must be digitally accessible and in English. 2) Regulations must be valid and adopted. 3) Recommendations and ethical advice must origin from collective entities of regulatory bodies or specialized entities.

The search of documents followed a search string in English as the official global language as well as the official and working language of the EU:

“artificial Intelligence + medicine + diagnosis + image” or

“artificial Intelligence + medical + diagnostic + imaging”

The search strings were entered into databases of global and intergovernmental organizations as well as of the EU and related collective entities (S1 Table). For intergovernmental organizations whose websites did not allow search strings to be entered into the database, all documents were searched individually by considering the search string. Then, relevant documents were identified. Documents that were marked as ineligible and had titles in languages other than English were removed. Next, documents were screened and appraised for eligibility and quality. Based on the title and the abstract or summarizing information, documents outside the scope of AI, medicine, diagnostics, imaging, ethical recommendations, and regulations were excluded. Documents that were not fully available were not further retrieved. The assessment for eligibility included a full document screening: Excluded were duplicates, repealed regulations, documents that were not related to AI image-based diagnostics, flagged as non-citable draft, and executive summaries available in a complete version. The screening process and criteria were discussed by three researchers and implemented in practice by one researcher. The data quality was appraised regarding the inclusion and exclusion criteria, objectivity, and the content relevant to the research object. The included regulations were examined on the availability of newer versions. Then, the latest version was chosen. After we completed searching, screening, and including official documents, text sections relevant to the research object were identified and extracted. Subsequently, further documents were identified through citation tracking (Fig 1).

**Fig 1:**
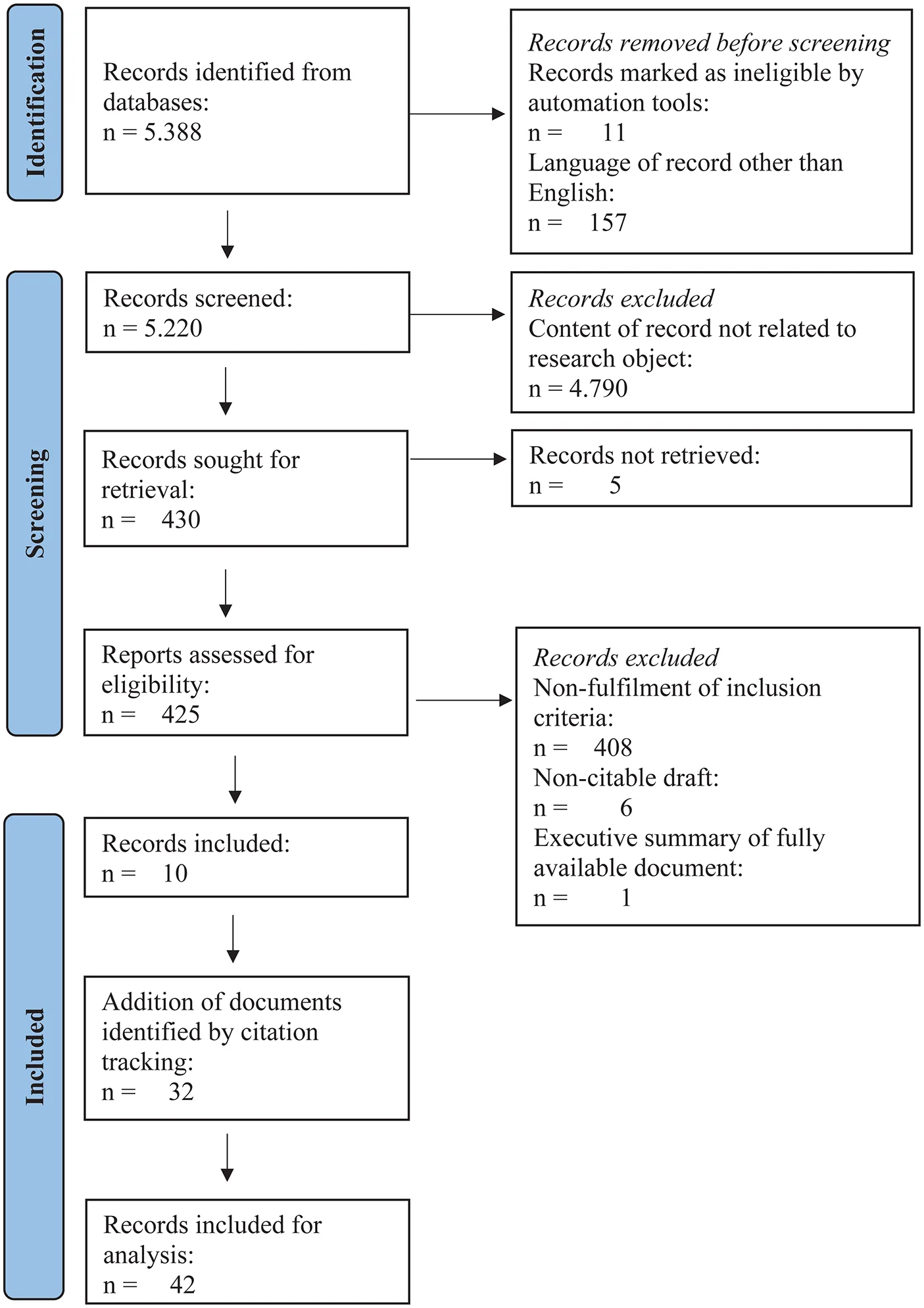
PRISMA flow diagram [13] of the search, screening, and inclusion process of the official documents.

We used backward citation tracking on the extracted text sections. The documents that we identified were evaluated by using similar inclusion and exclusion criteria. This process was repeated until we could not identify any further documents relevant to the research topic.

Data relevant to the research object (S2 Table) was extracted from the included documents and assigned to the categories of regulatory documents and ethical recommendations as guiding documents at the international and the EU level (Fig 2).

**Fig 2:**
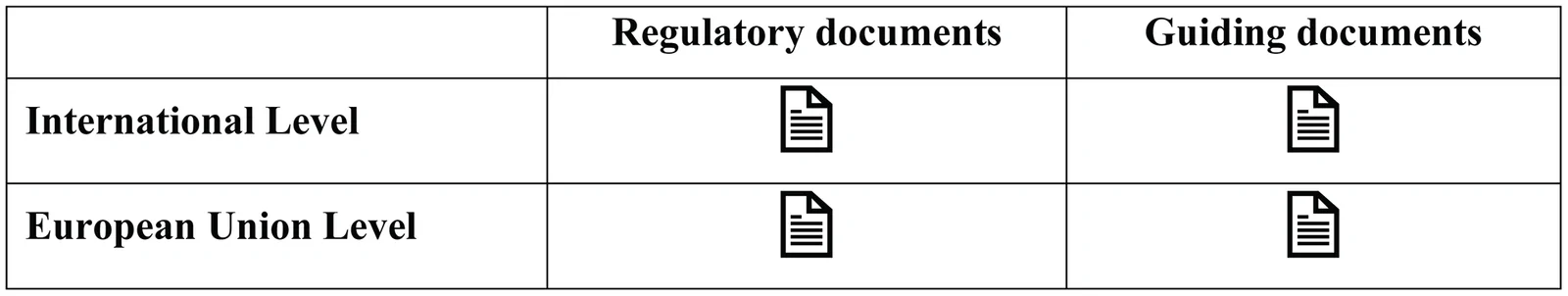
Representation of the categories used to assign extracted data.

The data included in the research was analyzed by using the qualitative content analysis [14] and thematic analysis [15,16]. Both methods are based on a qualitative analysis of text-based data and a formation of thematic categories. However, the qualitative content analysis is characterized by a highly structured methodical procedure, while the thematic analysis focusses on gaining in-depth meaning. Combining both methods allow gaining deeper insights and understanding of thematically structured data. At first, the extracted data was coded to detect the main thematic categories of the ethical and regulatory framework. Thereby, the extracted data read intensively and an umbrella term for each thematic section was written next to it. This procedure was applied to all data. Following, based on the codes, categories were formed inductively. The content was assigned accordingly before the categories were revised deductively following the thematic structures of the regulations. Next, the categorized data was analyzed within each individual level followed by comparing and contrasting the findings. The categories and findings were discussed by researchers (NP, MO, DB, FS) with technical expertise in medical and research ethics, legal and political studies, computational neuroscience, and medicine.

Finally, to ethically analyze these results, we conducted a principle-based approach by Beauchamp and Childress [17]. This approach is widely used as a core method in the field of medical and clinical ethics and enables the reasoning and analysis of ethical challenges according to four principles: autonomy, beneficence, non-maleficence, and (social) justice. The principle of autonomy refers to a medical decision-making process that considers individual preferences, and beneficence implies balancing patients’ potential risks against benefits. Non-maleficence emphasizes the prevention of harm, while the principle of (social) justice considers a fair distribution of resources [17]. Given the clinical application of the AI system in patient care, this approach is equally applicable to ethically analyzing medical AI systems. The ethical evaluation focusses mainly on patients. However, users of the medical system are considered when actions are contributing to the patients’ medical care.

## Results

The most important finding is that the ethical and regulatory framework of the behavioral performance of AI within the development and use for image-based diagnostics at the international and the EU level are primarily composed of broadly applicable regulations for general medical AI systems. The international regulation is limited to the International Covenant on Civil and Political Rights, a universal instrument of the United Nations, and only applicable to countries that have ratified this resolution. The guidance is mainly based on recommendations of international organizations. International guidance is predominantly recommended to its member states; however, all global states are encouraged to follow their advices.

We identified five thematic categories of the regulatory and ethical framework: (i) data basis, (ii) reliability, (iii) comprehensibility, (iv) disclosure, and (v) usability.

### Data basis

The framework of the data basis of the medical AI system is composed of data standardization, data assessment, and data quality (Table 1).

**Table 1:** Representation of the regulatory and guiding ethical framework on the data basis applicable for the behavioral performance of AI for image-based diagnostics on the international and EU level. [S2 Table: International Level/ Guidance: 2, 5, 10, 12; EU/ Regulations: 17, 20, 23; EU/ Guidance: 24, 27, 29-31, 34, 36, 40, 42].

		International Level	European Union
**Regulations**	Data Standardization		Standardized dataset catalogue
	Data Assessment		Data validation and testing
	Data Quality		Criteria-based quality, security measures
**Guidance**	Data Standardization	Data interoperability standards	Semantic interoperability
	Data Assessment	Data verification check	Critical data assessment
	Data Quality	Sufficient and representative data (quality)	Data integrity

#### Data standardization.

On the international level, introducing interoperability standards for health data is recommended. In the EU, by regulation, a standardized machine-readable data set catalogue shall be provided, and a semantic interoperability of health data is advised.

#### Data assessment.

Data verification checks are internationally recommended. In the EU, data must be validated, tested and is advised to be complemented by a critical assessment of data that is used.

#### Data quality.

On the international level, data quality should be sufficient and data representative for the intended purpose. In the EU, data that is used shall, by regulation, fulfill defined criteria, and appropriate security measures must be established against unauthorized access to destruct, modify or the loss of data. Moreover, it is recommended to ensure data integrity.

### Reliability

The framework for the reliability of the medical AI system is composed of assessment, the intended performance, robustness, accuracy, and compliance (Table 2).

**Table 2:** Representation of the regulatory and guiding ethical framework on reliability applicable for the behavioral performance of AI for image-based diagnostics on the international and EU level. [S2 Table: International Level/ Regulations: 1; International Level/ Guidance: 3, 10-13; EU/ Regulations: 14, 16-19, 21, 22; EU/ Guidance: 24-27, 29, 34, 37, 40, 42].

		International Level	European Union
**Regulations**	Assessment		Clinical evaluation, post-market monitoring
	Intended Performance	Right to security	Use instruction, capability of intended performance, (cyber-) security
	Robustness		Robustness
	Accuracy	Right to life, prohibition of inhuman treatment	Accuracy, accuracy metrics
	Compliance		Compliance, EU values, principles and interests
**Guidance**	Assessment	Performance testing	External auditability
	Intended Performance	Documenting purpose and functions, user information	Mitigating unintended use, intended behavior
	Robustness	Robustness	
	Accuracy	Responsibility for inaccuracies	Indicating inaccuracies
	Compliance	Ethical considerations, moral values	Principles, human rights and values by-design

#### Assessment.

Internationally is recommended to test the AI performance considering the intended use. In the EU, a clinical evaluation of the device and a post-market monitoring process is mandatory while an external auditability is recommended.

#### Intended performance.

The right to security is protected internationally. The intended purpose and function of the AI system should be documented and user information provided. In the EU, instructions for use must be developed and the system shall be capable of achieving the intended performance. Everyone’s right to security is protected and the AI system shall achieve an appropriate level of cybersecurity. The system is advised to reliably perform the intended behavior and prevent and mitigate unintended applications, such as dual-use.

#### Robustness.

Internationally, the AI system is advised to be robust, while in the EU, the AI system is regulatorily required to function appropriately through robustness.

#### Accuracy.

The regulatory frame of accuracy is internationally based on the inherent right to life and the prohibition of the subjection to inhuman or degrading treatment. Additionally, it is recommended to assume responsibility for inaccurate content. In the EU, an appropriate level of accuracy must be achieved and be disclosed combined with its metrics. Further, indicating the probability of inaccuracies is advised.

#### Compliance.

Internationally it is advised to integrate ethical considerations and moral values into the AI system’s design. In the EU, values, interests, and principles of the EU must be respected. The AI system’s use and development should be based on defined principles and consider human rights and values by-design.

### Comprehensibility

The framework of the comprehensibility of the medical AI system is composed of explainability, understandability, and traceability (Table 3).

**Table 3:** Representation of the regulatory and guiding ethical framework on comprehensibility applicable for the behavioral performance of AI for image-based diagnostics on the international and EU level. [S2 Table: International Level/ Regulations: 1; International Level/ Guidance: 3, 4, 10-13; EU/ Regulations: 18, 22; EU/ Guidance: 24, 26-28, 32, 34, 37, 41, 42].

		International Level	European Union
**Regulations**	Explainability	Right to freedom of thoughts, information, and opinions	Right to explanations
	Understandability		
	Traceability		Traceability
**Guidance**	Explainability	Capacity-, clinical- and science-based explanations	Explainable to humans
	Understandability	Providing understanding	Understandable information
	Traceability	Traceability on development stages	

#### Explainability.

On the international level, providing explainability is based on the right to freedom of thoughts, to hold opinions, and the right of freedom to receive information without any distinction. Explanations should be clinical and scientific taking into account the capacity of the recipients. In the EU, persons who receive decisions based on an AI output have the right to receive explanations. The provided information is recommended to be explainable to humans.

#### Understandability.

Internationally is recommended to establish an understanding of the functionality, the outcome, and decision-making to the variety of interest-holders. In the EU, understandable information on the system’s processes, output, decision-finding, its verification, and values should be provided.

#### Traceability.

Internationally is advised to provide traceability across development stages while in the EU, traceability throughout the AI system’s lifecycle must be provided.

### Disclosure

The framework of the disclosure of the medical AI system consists of transparency, operational disclosure, and collaborations (Table 4).

**Table 4:** Representation of the regulatory and guiding ethical framework on disclosure applicable for the behavioral performance of AI for image-based diagnostics on the international and EU level. [S2 Table: International Level/ Guidance: 2, 3, 6, 9-13; EU/ Regulations: 18, 21-23; EU/ Guidance:24, 26-29, 31, 32, 34, 35, 37, 40, 42].

		International Level	European Union
**Regulations**	Transparency		Transparency, disclosure of human-AI-interaction
	Operational Disclosure		Monitoring system operation
	Collaborations		
**Guidance**	Transparency	Transparency, disclosure of context-specifics and conflicts of interests	Sufficient information provision
	Operational Disclosure	Continuous operational disclosure	Information on reasoning underlying operations
	Collaborations	Disclosure of interests and parties involved	

#### Transparency.

On the international level, the decision-making process is recommended to be transparent, and context-specific information as well as conflicts of interest should be disclosed. In the EU, ensuring transparency is regulatorily required, and users must be informed when interacting with an AI-driven system. Further, health professionals and patients are advised to be sufficiently provided with information.

#### Operational disclosure.

Internationally is advised to continuously provide an operational disclosure. In the EU, system operations must be transparent and users are advised to be provided with information on the reasoning that is underlying the data processing operations of AI system.

#### Collaborations.

On the international level, developing and deploying AI systems within public-private partnerships, commercial interests or collaborations with commercial parties should be disclosed.

### Usability

The framework of the usability of the medical AI system is composed of resources and competency (Table 5).

**Table 5:** Representation of the regulatory and guiding ethical framework on usability applicable for the behavioral performance of AI for image-based diagnostics on the international and EU level. [S2 Table: International Level/ Regulations: 1; International Level/ Guidance: 2-8, 10-12; EU/ Regulations: 15, 19, 23; EU/ Guidance: 25-28, 32-34, 36, 37, 39, 42].

		International Level	European Union
**Regulations**	Resources	Right to receive information	Providing Union language
	Competency		Right to education, training, AI literacy
**Guidance**	Resources	Considering infrastructure and users’ needs	
	Competency	(AI) Education, user’s capabilities as part of the performance	Up- and re-skilling, education on cybersecurity

#### Resources.

On the international level, everyone is protected by the right of freedom to receive information without distinction, such as language. It is advised to consider the health informatic infrastructure as a factor on the clinical performance and to describe how the system responds to user’s needs. In the EU, the medical device must be available in an official Union language.

#### Competency.

Providing (AI) education is internationally recommended. The user’s capabilities should be considered in the clinical performance of the medical system, and persons developing and using the system should receive an adequate training. In the EU, everyone has the right to education and access to continuous learning, in particular to training when introducing a new technology. Providing sufficient AI education is regulatorily obligated and patients must be supported in developing relevant competences. Education on cybersecurity as well as up- and re-skilling is advised.

## Discussion

The regulatory and ethical framework of the behavioral performance of medical AI for image-based diagnostics on the international and EU level are ethically discussed considering the principles by Beauchamp and Childress [17]: autonomy, beneficence, non-maleficence, and (social) justice [17] (Fig 3).

**Fig 3:**
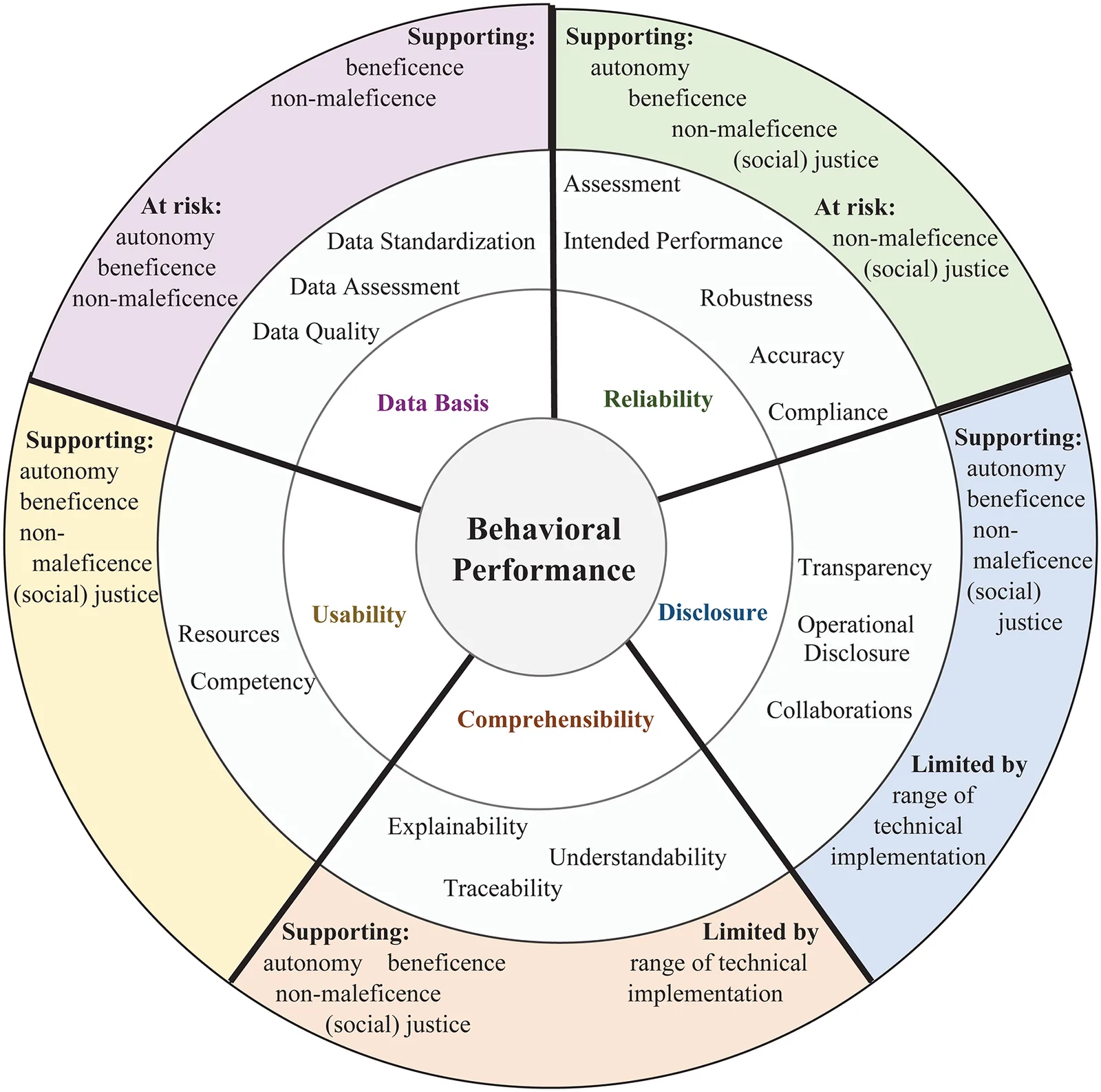
Representation of ethical challenges of the regulatory and ethical framework of the behavioral performance of medical AI systems for image-based diagnostics on the international and EU level.

Despite particular challenges and characteristics of medical AI systems for image-based diagnostics, its regulatory and ethical framework on the international and EU level is equal to the framework of general medical AI systems [18–20]. Common international and EU regulations of specifically image-based diagnostic AI systems as a basis for a cross-border development, global market, and use of image-based diagnostic systems are currently insufficient.

### Data basis

The data basis of image-based medical AI diagnostic systems is internationally not regulated, but guided by an ethical framework. This framework allows states and AI actors to voluntarily follow recommendations in order to improve the framework of the data basis of image-based AI systems. However, to sufficiently protect the data basis as an essential element of the AI system and to improve data standardization not only within the EU, but across international borders, international-wide regulations are needed.

#### Data standardization.

Establishing data interoperability standards is recommended on the international level, complemented by advising semantic interoperability in the EU and could enhance data quality, facilitate a broader data use, and significantly improve the AI performance [21]. Improving data standardization and the conditions for an extensive image data use could enhance the accuracy of diagnostic results and thereby support the principle of beneficence.

In the EU, providing a standardized machine-readable data set catalogue is mandatory. Creating machine-readable datasets enables an automatic data analysis and could enhance an appropriate data use [22]. Improving the use of image data as a basis for appropriate diagnostic results and medical decision-making could support the principle of beneficence.

#### Data assessment.

Data verification checks are internationally recommended and enable an examination of the completeness, accuracy, and compliance of data sets by focusing on data performance [23].

Additionally, data validation and testing are regulatorily required in the EU and complemented by a recommendation for critical data assessment. Data validation targets the purpose of the data and enables an improvement of the overall data quality. By analyzing data sets, errors in the training data can be detected and the accuracy and quality of the AI model can be improved [23–25].

Data assessments could improve the data quality and enhance the reliability of the foundation of the medical decision-making. Thereby, the adequacy of therapeutic interventions could improve and strengthen the principle of beneficence. Responding to identified errors early in the AI lifecycle could decrease the risk of inaccurate image-based diagnostic results and false diagnoses and thereby support the principle of non-maleficence.

#### Data quality.

On the international level, a sufficient data quality and the use of representative data are recommended while in the EU, data quality must follow criteria. The quality of data sets used for AI systems is a significant factor of an effective algorithm [26] and a reliable performance of the model [27]. Providing high quality and purpose-based data for the development could improve the effectiveness and reliability of the diagnostic system. The quality of diagnostic results as a foundation for an effective therapeutic intervention could improve and strengthen the principle of beneficence. New approaches to improve the quality of medical images are developed [28]. However, medical image data maintains the challenge of non-pathological information being encoded and affecting the data quality and performance of the system. Such factors include information on scanning devices that vary according to manufacturers and follow different scanning protocols [29,30]. The level of noise varies according to scan devices and artifacts could result from erroneous image-data processing [31]. Circumstances of scanning patients, such as radiation doses, contrast, and resolutions of images could severely impact and decrease the data quality [31]. Based on the high variability of particularly medical image data contributing to a lower data quality and decreasing model performance [5], challenges arise in the implementation of the regulations and recommendations.

In the EU, security measures against unauthorized access to manipulate data must be established and data integrity should be provided. Protecting data security that supports the maintenance of data integrity could lower the risk of false data use. Lowering the number of false data as a basis for diagnostics could decrease misdiagnosis. Thus, the risk of incorrect medical treatment could decrease and support the principle of non-maleficence. Due to the complexity of medical image data, image-based diagnostic systems are highly susceptible to adversarial attacks that could enable an unauthorized access [32]. A previous study has proved that adversarial attacks enable the alteration of medical image data. In this study, researchers simulated an adversarial attack that modified diagnosis-sensitive contents of mammogram images [33]. The manipulated image data could mislead the diagnostic AI model and cause incorrect breast cancer diagnoses [33].

Further, manipulation through data poisoning could affect the learning process of the AI model and produce biased results that could risk discrimination against groups of humans [32,34]. In response to data poisoning, numerous defense mechanisms have been developed [32] and are continuously competing against the development of however more advances malicious systems [35,36]. Establishing security measures that prevent unauthorized data access and manipulation of data remains challenging [37].

Using diagnostic results for medical decision-making that potentially develop from altered data inadequate for individual patients could risk biased decision-making. Considering patients making decisions that are based on incorrect information could jeopardize the principle of autonomy. Further, inaccurate diagnostic results could lead to mistreating patients and violate the principles of beneficence and non-maleficence.

Considering the rapid development of technology, establishing regulations is currently not sufficient to guarantee cybersecurity measures that are necessary to protect the data basis against malicious data alteration. However, the consideration of the systems’ challenges, such as a high variability of image data and susceptibility to cybersecurity that risks data manipulation could improve data quality. Establishing regulatory measures on the international level is essential to protect a global cross-border protection of medical image data.

### Reliability

#### Assessment.

Performance testing is internationally recommended, enables an evidence-based evaluation of the system’s accuracy as well as effectiveness and requires the consideration of the diagnostic AI system’s specifics [38].

In the EU, clinical evaluation and post-market monitoring are mandatory and external auditability is recommended. Evaluating the clinical performance of the AI system allows an assessment of the algorithmic performance with respect to the diversity of humans [39], but its effectiveness depends on the chosen method [39]. Post-market monitoring allows the identification of outcomes and potential errors that were not identified in pre-market evaluations [40]. However, despite various monitoring approaches, current methods do not enable a fully reliable evaluation of the AI performance [41].

Assessments to test and monitor the performance of image-based diagnostic AI systems at different stages of the AI lifecycle could reduce the risk for diagnostic errors. Diagnostic results could improve, enable the consideration of adequate therapeutic options, and thereby strengthen the principles of beneficence and non-maleficence. However, relying on an obligated, but in effectiveness limited evaluation could lead to developing blind trust in the diagnostic system. Incorrect results could remain undetected, lead to a medical decision-making that would not fully consider the individual’s needs and thereby challenge the principle of autonomy.

#### Intended performance.

Providing user information and to document the intended purpose and functions are internationally advised. In contrast, in the EU, developing use instructions is mandatory. Developing use instructions could support users to understand the functionality of the diagnostic system, which is a foundation for reaching an intended use. In the EU, the AI system must be capable to achieve the intended performance. Achieving an intended performance of an AI-driven analysis of medical images, enables a precise, personalized and quick diagnoses [4], could save time and enhance the decision-making process. Further, the rate of errors in analyzing medical images caused by human factors could decrease [42,43]. However, factors, such as prejudice or discrimination that are considered to be human, are equally risks of image-based AI systems.

Non-pathological demographic factors of patients, such as age, sex or race, are factors that could be considered in AI image-based diagnostic systems [44]. A research team conducted a study on chest X-ray image data across the fields of radiology, dermatology, and ophthalmology and could prove that diagnostic image-based AI systems used demographic shortcuts when classifying different diseases [44]. As a part of the performance, models encoded demographic information by algorithms and used shortcuts to identify diseases risking unfair predictions [44].

Providing patients with decision-making options based on adequate diagnostic results could strengthen the principle of autonomy. Precise diagnostic results could improve patients’ medical outcome as well as saving time resources that could be spent for treating further patients. Thereby, the principles of beneficence and (social) justice could be supported.

However, achieving the intended performance of image-based medical AI systems is challenged by multiple factors, such as the use in real-world conditions. For example, the performance of image-based diagnostic AI systems is related to scanner-specific factors and the risk of scanner domain shifts [29]. Moreover, image-based AI systems have a lower performance rate when being used in real-world settings; in particular when medical images from hospitals or scanner manufacturers were not considered in the training of the AI system [29]. The challenge of cross-hospital data shift of medical imaging has been outlined in a previous study of an image-based AI systems for the diagnosis of COVID-19 [45]. Researcher found out that the AI system’s high accuracy would depend on dataset-specific text markers and patient positioning instead of pathology [45]. Its diagnostic performance on COVID-19 significantly decreased when it was used in another hospital setting [45]. In addition to scanning equipment, the department in which the scanner is used could impact the prevalence of same diseases, cause sampling biases, and cause false correlations in the diagnostic system [30]. For instance, the number of certain diseases that are detected on images from portable scanners in intensive care differs from the diseases used by scanners in inpatient units. Consequently, the probability of patients receiving a positive AI-based diagnosis correlates to the probability of diseases that were frequently detected in the scanner’s unit; irrespective of pathological components of individual patients [30].

On the international level and in the EU, everyone is protected by the right to security. In the EU, an appropriate level of cybersecurity must be achieved. Image-based models possess particular vulnerabilities to cybersecurity risks, such as adversarial and service denial attacks that risk the system’s availability and false diagnostic results [46,47]. A team of researchers has proved this vulnerability by developing an AI-based model for detecting COVID-19 pneumonia based on X-ray images [48]. After conducting an adversarial attack, the accuracy of the model’s diagnosis reduced from 96.51% to 10.13% [48]. As a result, an image free from pneumonia was misclassified as a COVID-19 pneumonia diagnosis with a confidence of 99.94% [48].

While the resistance to attacks can be improved, the dynamic of a continuously adaptation and development of defenses against new cyber threats challenges effective security against cyber threats [47,49]. This dynamic process results in an insufficient security provision and a yet unforeseeable risk potential. Considering this unknown risk potential and the unforeseeable future development of technical possibilities of a medical high-risk diagnostic system, the establishment of an adequate and obligatory framework is essential to protect the intended performance.

In the EU, the potential for an unintended application of the AI system should be mitigated and prevented. While cyberattacks enable an unintended misuse [34], AI is considered as a dual-use technology with capabilities for known and arising threats [50] in unprecedented dimensions. The risk of using the diagnostic system with unpredictable harmful outcomes for individuals and the society could conflict with the principle of non-maleficence.

The provision of cybersecurity requires a cross-border international cooperation of countries to effectively provide protection against cyber threats [51]. A normative approach that advises countries to voluntarily cooperate against international structures of cybercrime is not sufficient. Instead, a harmonized international regulatory framework that standardizes security is necessary for obligating countries to cooperate [51]. Protecting the security of individuals from cyber threats could support the principles of beneficence and non-maleficence by preventing malicious impacts that risk inaccurate diagnostic results and improve effective therapeutic interventions. However, cybersecurity cannot be fully ensured. A medical AI system capable of an intelligent analysis of image data that is vulnerable to cyber threats without reliable defense mechanisms, poses risks to the right to security and the maintenance of a safe and secure medical system. The use of an AI diagnostic system that is potentially manipulated and follows an unintended performance could cause incorrect diagnostic results and risk falsely indicated or the withholding of diagnostics or medical treatment. Thereby the principle of non-maleficence could be jeopardized. Allocating resources to unnecessary medical treatments would conflict with the principle of (social) justice. The current framework does not effectively protect the intended performance of image-based diagnostic AI systems. A consideration of the systems’ challenges, such as the models’ vulnerabilities to cyber security risks is necessary to improve the regulatory protection of the AI systems’ intended performance.

#### Robustness.

An appropriate function through robustness is internationally recommended, while in the EU, the AI system must be robust. The robustness of the medical imaging model can be improved, significantly increase the resistance to noise and adversarial attacks and thereby improve the maintenance of its functionality [47,52]. Increasing the robustness could decrease the risk of erroneous diagnostic results, improve patients’ medical treatments and therefore strengthen the principles of beneficence and non-maleficence. However, the particular characteristics of medical imaging challenge the robustness against the diversity of perturbation and real-world conditions [47,52]. The term robustness allows different interpretations across domains and comprises a variety of concepts [12] risking a globally divided interpretation of the regulatory and ethical framework.

#### Accuracy.

On the international level, humans are protected by the right to life and against being subjected to inhuman or degrading treatment. Medical AI systems that do not affect or limit patients’ life expectancies and protect patients from degrading treatments through accurate AI results could improve diagnostics and support the principles of beneficence and non-maleficence.

In the EU, the AI system must achieve an appropriate level of accuracy and disclose its information combined with the accuracy metrics. The accuracy of an AI system depends on various factors, such as the data basis, its application, cyber threats, and time and requires appropriate measures [26,52,53]. Accuracy metrics provide insights into the correctness of a system’s outcomes, influence its classification and subsequent decision-making processes [54]. An appropriate accuracy level could improve diagnostic results and strengthen the principle of beneficence. However, the quality and reference standards used to calculate the metric varies and challenges the interpretation of the metric including in form of an overestimating values and misinterpretations could arise [54].

#### Compliance.

Integrating ethical considerations and values into the AI system is internationally advised. In the EU, compliance with law, values, and principles is mandatory and complemented by the advice to respect AI principles and values. Compliance and the integration of values and principles set a framework for responsibly protecting patients’ well-being, minimizing harm, and implementing technology align with human values [55]. Developing and using a compliant and value-based diagnostic system could enforce the principles of autonomy, beneficence, non-maleficence, and (social) justice.

### Comprehensibility

#### Explainability.

On the international level, everyone is protected by the right to freedom of thought, to receive information, and to hold opinions. AI systems have the potential to strengthen the opinion-making process and to adjust human thoughts and opinions [56]. Providing sufficient information on the AI system’s results supports physicians and patients to form individual opinions on the diagnostic results [56]. Supporting an independent medical decision-making could strengthen the principle of autonomy.

Further, clinical and scientific explanations are internationally recommended while in the EU, explanations are mandatory and should be provided to humans. Explainability enables physicians to gain a deeper understanding of the system’s behavior and to better evaluate the diagnostic system [6]. Evaluating the decision-making process could improve an autonomous medical decision-making, protect patients from misdiagnosis, and strengthen the principles of autonomy and non-maleficence.

The meaning of explainability is not universally agreed upon and the techniques for explainable AI-solution vary in usefulness, complexity, and accordance with the clinical decision-making process of physicians [6,57]. In particular medical image-specific characteristics and the use of black box models challenge the extent of explainability that is necessary for useful clinical decision-making [6]. To explain the AI decision-making to physicians, visual explanations as heatmap-based approaches are commonly used and received as trustworthy and reliable information [58]. However, heatmaps are sensitive to spurious correlations, such as scanner-specific artifacts that correspond to labels in training data [58]. Physicians could create a false impression to receive pathological relevant information and risk an incorrect medical decision-making.

#### Understandability.

Internationally, information on the system should be sufficiently understandable for interest-holders while in the EU, understandable information on the AI specifics should be provided. Understanding the explanations of the AI system increases the clinical confidence of physicians [6]. By providing understandable explanations, the physician’s clinical confidence could enhance the quality of diagnostics, the trust in the decision-making process and thus, support the principles of autonomy and beneficence.

#### Traceability.

While providing traceability is internationally recommended, it is mandatory in the EU. Traceability is considered as a precondition for explainability and provides information on the system’s operation that are otherwise limited by the characteristics of black box models [59,60]. Thereby, the system’s behavior could be audited and anomalies could be detected [59,60]. Providing traceability could improve transparency on decision-making, provide patients information that are essential as a basis of an informed decision-making and thereby strengthen the principle of autonomy. Traceability is a basis for improving human oversight, identifying inconsistencies, and improving the overall quality of the image-based diagnostic performance. Thereby, physicians could be supported to evaluate and improve diagnostic results and the principles of beneficence and non-maleficence could be strengthened. However, the variety of models supporting traceability varies in effectiveness and the complexity of the system’s model, inconsistencies, and data quality challenges the traceability of diagnostic systems [59].

### Disclosure

The disclosure of image-based AI systems for diagnostic purposes is internationally not regulated. Instead, AI systems are guided by ethical recommendations. In contrast, the EU has developed regulations that are extended by ethical guidance. Missing international regulations allow states and developers to voluntarily follow ethical guidance on disclosing information on medical systems that are classified as high-risk.

#### Transparency.

Transparency on the decision-making of the AI system, its context-specifics, and conflicts of interests are internationally advised. In the EU, ensuring transparency and informing users about interacting with AI is obligated. Providing transparency of the image-based AI system could enhance the understanding of the model’s output, the reliability of the system, and building trust that further enables a responsible clinical application [7]. Improving the application of the system as well as the detection of bias through transparency could improve the circumstances of a patient-centered decision-making and support the principle of autonomy. The identification of bias is essential for the awareness and prevention of biased diagnosis and improvement of medical treatments. Therefore, the principles of beneficence and non-maleficence could be supported. Despite multitude approaches to improve transparency in varying degrees, deep learning systems used to develop AI image-based diagnostic systems are limited in their interpretability and explainability [7].

#### Operational disclosure.

Continuous operational disclosure is internationally recommended, while in the EU, monitoring the system’s operation is mandatory. Providing transparency on the system’s operation requires traceability and enables an evaluation of the operational information on the processes concluding in diagnostic results [59,60]. Verifying the operational processes could strengthen the system’s accountability and improve assessments [60]. Subsequently, errors could be identified, diagnostic results enhance, and thereby serve as a foundation for a transparent decision-making. Thus, the principles of autonomy, beneficence, and non-maleficence could be supported. However, providing operational disclosure is limited by traceability.

#### Collaborations.

The disclosure of public-private partnerships, commercial interests, and collaborations is internationally recommended. Developing AI systems within public-private partnerships and collaborations risks an imbalance of power between the parties involved and foster a dependent relationship with potentially conflicting interests [61]. The use of deep learning systems limits the provision of information on manipulation and data used that influences the system’s performance [61]. Disclosing collaborations could improve transparency regarding potential commercial interests that influence the development and decision-making of the medical AI systems and thereby support the principles of autonomy and (social) justice.

### Usability

#### Resources.

Internationally, everyone is protected by the right to receive information without any distinction. Further, the user’s needs and the infrastructure should be considered when developing AI systems. In the EU, providing an official Union language is mandatory. To establish usable medical systems, physicians require understandable information that consider physicians’ needs, including abilities and disabilities. Considering user-oriented languages in textual information and visual impairments in visual information [62] could support physicians to understand necessary information for using the AI system. Protecting the right to information through minimizing barriers could facilitate the use of the system, decrease the risk of an incorrect use, and could thereby support the principles of autonomy, non-maleficence, and (social) justice.

Adequate infrastructural conditions are essential to provide usability, an integration of the image-based AI system into the clinical workflow, and to improve the diagnostic performance [63], while constraints could significantly restrict the access and use [64]. Considering the condition of the infrastructure in the development process could support image-based AI systems in achieving their intended function, improve diagnostics and support the principle of beneficence.

#### Competency.

Providing education and considering the users’ capabilities in the clinical performance is internationally advised. In the EU, the right to education and continuous learning is protected and patients must be supported in receiving relevant competencies. Additionally, education on cybersecurity and an up- and re-skilling are recommended. The user’s skills influence the potential benefits and harms of the AI system and thus affect its clinical performance [65]. Providing AI education on medical systems enhances users and developers to perceive the system’s strengths and limitation, to evaluate the system’s results, and to detect potential bias and errors [66]. A continuous learning is required to keep up with technical developments and to adapt competencies accordingly [65]. Educating patients could empower participation in medical decision-making, improve the patient-physician relationship and the overall patient’s well-being [67].

Improving AI literacy could enhance the performance and clinical application of the diagnostic AI system. Harmful impacts could be reduced, patient participation in medical decision-making could improve and thereby strengthen the principles of autonomy, beneficence, and non-maleficence.

Particular challenges and characteristics of image-based diagnostic AI systems, such as vulnerabilities to cyber threats are equal challenges in further medical AI systems [68]. Considering that the sufficiency of the ethical and regulatory framework corresponds to the individual technical characteristics of AI system, further medical AI systems’ performances could be insufficiently protected on the international and EU level.

In conclusion, the ethical and regulatory framework of the behavioral performance of image-based diagnostics AI systems on the international and EU level can be categorized into data basis, reliability, comprehensibility, disclosure, and usability. Thereby the ethical principles of autonomy, beneficence, non-maleficence, and (social) justice could be supported. However, similar principles could be challenged by the range of technical implementations.

Despite particular characteristics and challenges of image-based diagnostic AI systems, its behavioral performance is protected by similar regulations as general medical AI systems. These allow a wide range of technical measures to ensure compliance, however the effectiveness of these technical approaches varies significantly. Considering the universal disagreement on technical terminology, the varying effectiveness of technical approaches, and the rapid development of technical possibilities, the regulatory and ethical framework for this particular diagnostic AI system can currently not sufficiently protect individuals and the society. International and EU ethical guidance could support the voluntary integration of protective instruments into the development and use of medical AI systems for image-based diagnostics and serve as a basis for legislative procedures. However, to obligate countries and developers to follow a protective framework and to effectively protect the AI system beyond voluntary action, an international regulatory frame is essential.

Therefore, we recommend enacting a standardized regulatory framework for image-based diagnostic AI systems that responds to the challenges the systems’ characteristics. Appropriate security measures should be internationally adapted that specifically focus on protecting data and the intended performance adequately. Further, the wide range of technical measures that allow a compliant but insufficient effectiveness, should be limited.

The technical characteristics of medical AI systems could affect the sufficiency of the ethical and regulatory framework for the AI behavioral performance. Therefore, we suggest further research on the international and EU regulatory and ethical framework of the behavioral performance of other characteristics of medical AI systems. Based on the research focus on the behavioral performance on the international and EU level, we further advise to extend our research by determining other thematic categories and examining the regulatory and ethical framework on national levels.

## Supporting information

S1 Table Databases of global and intergovernmental organizations. (DOCX)

S2 Table Regulative and guiding documents included into the research. (DOCX)
