# Supplementary material for: The ethical and regulatory framework of medical artificial intelligence (AI) system’s behavioral performance for image-based diagnostics: An ethical evaluation of the International and European Union Scope
Source: PLOS Digit Health. 2026 Oct 14;5(10):e0001781. doi: 10.1371/journal.pdig.0001781 (PMC13667779; doi:10.1371/journal.pdig.0001781)
Supplement: Supplementary file 1 [file pdig.0001781.s001.docx]

**S1 Table**

| **Databases** | **Website** | **Search Date** |
| --- | --- | --- |
| **International Level** | | |
| Organisation for Economic Co-operation and Development | https://www.oecd.org/content/oecd/en/search/publications.html | May 7th, 2025 |
| UNESCO | https://unesdoc.unesco.org/advancedSearch/:new | April 29th, 2025 |
| United Nations | https://digitallibrary.un.org | April 29th, 2025 |
| World Health Organization | https://iris.who.int/discover?scope=%2F&query=&submit= | April 30th, 2025 |
| **European Union Level** | | |
| Alliance for Internet of Things Innovation | https://aioti.eu/category/publications/ | May 8th, 2025 |
| EUR-Lex | https://eur-lex.europa.eu/homepage.html?lang=de | May 9th, 2025 |
| European Commission | https://dorie.ec.europa.eu/en/home | May 9th, 2025 |
| European Court of Human Rights/ HUDOC | https://hudoc.echr.coe.int/eng#{%22documentcollectionid2%22:[%22GRANDCHAMBER%22,%22CHAMBER%22]} | May 9th, 2025 |
| European Data Protection Board | https://www.edpb.europa.eu/our-work-tools/consistency-findings/opinions_de?page=0 | May 8th, 2025 |
| Federation of European Academies of Medicine | https://www.feam.eu/feam-publications/ | May 8th, 2025 |
| Healthcare Data Innovation Council | https://healthdatainnovation.eu/white-paper/ | May 8th, 2025 |
