# Supplementary material for: The ethical and regulatory framework of medical artificial intelligence (AI) system’s behavioral performance for image-based diagnostics: An ethical evaluation of the International and European Union Scope
Source: PLOS Digit Health. 2026 Oct 14;5(10):e0001781. doi: 10.1371/journal.pdig.0001781 (PMC13667779; doi:10.1371/journal.pdig.0001781)
Supplement: Supplementary file 2 [file pdig.0001781.s002.docx]

**S2 Table**

| **International Level** | | | | | | | |
| --- | --- | --- | --- | --- | --- | --- | --- |
| **Type of Effect** | **Citation No.** | **Name of the Document** | **Type of Inclusion** | **Year** | **Issuing Authority** | **Application Area** | **Applicable Subjects** |
| **Regulative Documents** | 1 | International Covenant on Civil and Political Rights, 19 December 1966, 999 UNTS 171, Can TS 1976 No 47 (entered into force 23 March 1976) [ICCPR]. | Citation tracking | 1966 | UN General Assembly | Regions that have ratified the ICCPR | Reliability, comprehensibility, usability |
| **Guiding Documents** | 2 | Amnesty International, & Access Now. The Toronto Declaration: Protecting the right to equality and non-discrimination in machine learning systems. 2018. Available from: https://www.amnesty.org/en/documents/pol30/8447/2018/en/ | Citation tracking | 2018 | Amnesty International, Access Now | Global | Data basis, disclosure, usability, |
|  | 3 | G20. Ministerial Statement on Trade and Digital Economy. Tsukuba, Japan, 2019. Available from: https://www.mofa.go.jp/files/000486596.pdf | Citation tracking | 2019 | G20 Trade Ministers, Digital Economy Ministers | G20 member states | Reliability, disclosure, comprehensibility, usability |
|  | 4 | OECD, Recommendation of the Council on Artificial Intelligence, OECD/LEGAL/0449. | Citation tracking | 2024 | OECD | Member countries of the OECD | Comprehensibility, usability |
|  | 5 | OECD, Recommendation of the Council on Health Data Governance, OECD/LEGAL/0433. | Citation tracking | 2025 | OECD | Member countries of the OECD | Data basis, usability |
|  | 6 | UNESCO. Recommendation on the Ethics of Artificial Intelligence. Paris: UNESCO. 2022. Available from: https://unesdoc.unesco.org/ark:/48223/pf0000380455 | Citation tracking | 2022 | UNESCO | Member states of UNESCO, all AI actors | Disclosure, usability |
|  | 7 | UNESCO. Report of the IBC on Big Data and Health. Paris: UNESCO. 2017. SHS/YES/IBC-24/17/3 REV.2. Available from: https://unesdoc.unesco.org/ark:/48223/pf0000248724.locale=en | Citation tracking | 2017 | UNESCO/ IBC | Member states of UNESCO | Usability |
|  | 8 | UN General Assembly, Universal Declaration of Human Rights, 217 A (III). 1948. Available from: https://www.refworld.org/legal/resolution/unga/1948/11563 | Citation tracking | 1948 | UN General Assembly | Global | Usability |
|  | 9 | United Nations Educational, Scientific and Cultural Organization (UNESCO). Universal Declaration on Bioethics and Human Rights. Paris: UNESCO. 2005. Available from: https://www.unesco.org/en/legal-affairs/universal-declaration-bioethics-and-human-rights | Citation tracking | 2005 | UNESCO | Member states of UNESCO, globally recommended | Disclosure |
|  | 10 | World Health Organization. Ethics and governance of artificial intelligence for health: Guidance on large multi-modal models (LMMs). 2024. Available from: https://www.who.int/publications/i/item/9789240084759 | Seed document | 2024 | WHO | Member states of the WHO | Data basis, reliability, disclosure, comprehensibility, usability |
|  | 11 | World Health Organization. Ethics and governance of artificial intelligence for health: WHO guidance. World Health Organization. 2021. Available from: https://iris.who.int/handle/10665/341996 | Seed document | 2021 | WHO | Member states of the WHO | Reliability, disclosure, comprehensibility, usability |
|  | 12 | World Health Organization. Regulatory considerations on artificial intelligence for health. 2023. Available from: https://iris.who.int/handle/10665/373421 | Seed document | 2023 | WHO | All relevant stakeholders | Data basis, reliability,  disclosure, comprehensibility, usability |
|  | 13 | World Health Organization. WHO Consultation Towards the Development of guidance on ethics and governance of artificial intelligence for health. Geneva, Switzerland, 2–4 October 2019  Meeting report. Available from: https://iris.who.int/handle/10665/340089 | Citation tracking | 2019 | WHO | Member states of the WHO | Reliability, disclosure, comprehensibility |
| **European Union Level** | | | | | | | |
| **Regulative Documents** | 14 | Conference of the Representatives of the Governments of the Member States, Consolidated version of the Treaty on European Union, 2008/C 115/01, European Union (EU), 13 December 2007. | Citation tracking | 2008 | Representatives of the Governments of the Member States of the European Union | Member states of the European Union | Reliability |
|  | 15 | Council Directive of 12 June 1989 on the introduction of measures to encourage improvements in the safety and health of workers at work (89/391/EEC) (OJ L 183 29.6.1989, p. 1). | Citation tracking | 1989 | Council of the European Union | Member states of the European Union | Usability |
|  | 16 | Council of Europe (COE), European Convention on Human Rights, as amended by Protocols Nos. 11, 14 and 15, ETS No. 005, 4 November 1950. | Citation tracking | 1950 | Council of Europe | Member states of the Council  of Europe | Reliability |
|  | 17 | Council of Europe. Convention 108 + Convention for the protection of individuals with regard to the processing of personal data. 2018. Available from: https://rm.coe.int/convention-108-convention-for-the-protection-of-individuals-with-regar/16808b36f1 | Citation tracking | 2018 | Council of Europe | Member states of the Council of Europe, other signatory states | Data basis, reliability |
|  | 18 | European Commission. Commission Implementing Decision of 22.5.2023 on a standardisation request to the European Committee for Standardisation and the European Committee for Electrotechnical Standardisation in support of Union policy on  artificial intelligence. Brussels, 22.5.2023 C(2023) 3215 final. | Citation tracking | 2023 | European Commission | Member states of the European Union | Reliability, disclosure, comprehensibility |
|  | 19 | European Union (EU), Charter of Fundamental Rights of the European Union, 2012/C 326/02, 14 December 2007. | Citation tracking | 2012 | European Parliament, Council of the European Union, European Commission | Member states of the European Union, institutions and bodies of the European Union | Reliability, usability |
|  | 20 | Regulation (EU) 2016/679 of the European Parliament and of the Council of 27 April 2016 on the protection of natural persons with regard to the processing of personal data and on the free movement of such data, and repealing Directive 95/46/EC (General Data Protection Regulation). | Citation tracking | 2016 | European Parliament, Council of the European Union | Member states of the European Union | Data basis |
|  | 21 | Regulation (EU) 2017/745 of the European Parliament and of the Council of 5 April 2017 on medical devices, amending Directive 2001/83/EC, Regulation (EC) No 178/2002 and Regulation (EC) No 1223/2009 and repealing Council Directives 90/385/EEC and 93/42/EEC. | Citation tracking | 2017 | European Parliament, Council of the European Union | Member states of the European Union | Reliability, disclosure |
|  | 22 | Regulation (EU) 2024/1689 of the European Parliament and of the Council of 13 June 2024 laying down harmonised rules on artificial intelligence and amending Regulations (EC) No 300/2008, (EU) No 167/2013, (EU) No 168/2013, (EU) 2018/858, (EU) 2018/1139 and (EU) 2019/2144 and Directives 2014/90/EU, (EU) 2016/797 and (EU) 2020/1828 (Artificial Intelligence Act). | Seed document | 2024 | European Parliament, Council of the European Union | Member states of the European Union | Reliability, disclosure, comprehensibility |
|  | 23 | Regulation (EU) 2025/327 of the European Parliament and of the Council of 11 February 2025 on the European Health Data Space and amending Directive 2011/24/EU and Regulation (EU) 2024/2847. | Citation tracking | 2025 | European Parliament, Council of the European Union | Member states of the European Union | Data basis, disclosure, usability |
| **Guiding Documents** | 24 | A comprehensive European industrial policy on artificial intelligence and robotics. European Parliament resolution of 12 February 2019 on a comprehensive European industrial policy on artificial intelligence and robotics (2018/2088(INI)) (2020/C 449/06). | Seed document | 2019 | European Parliament | Member states of the European Union, European Commission | Data basis, reliability, disclosure, comprehensibility |
|  | 25 | Alliance for IoT and Edge Computing Innovation (AIOTI). AI for better health, AIOTI WG Health White Paper, Release 1.0. 2022. Available from: https://aioti.eu/wp-content/uploads/2022/12/AIOTI-White-Paper-AI-for-Better-Health-Final.pdf | Seed document | 2022 | AIOTI | Member states of the European Union | Reliability, usability |
|  | 26 | Artificial intelligence in a digital age. European Parliament resolution of 3 May 2022 on artificial intelligence in a digital age (2020/2266(INI)) (2022/C 465/06). | Seed document | 2022 | European Parliament | Member states of the European Union, Council of Europe, European Commission | Data basis, reliability, disclosure, comprehensibility, usability |
|  | 27 | Commission Staff Working Document Impact Assessment Accompanying the Proposal for a Regulation of the European Parliament and of the Council - Laying Down Harmonised Rules on Artificial Intelligence (Artificial Intelligence Act) and Amending Certain Union Legislative Acts. {COM(2021) 206 final} - {SEC(2021) 167 final} - {SWD(2021) 85 final}. | Seed document | 2021 | European Commission | Member states of the European Union | Data basis, reliability, disclosure, comprehensibility, usability |
|  | 28 | Council of Europe Commissioner for Human Rights. Unboxing artificial intelligence: 10 steps to protect human rights. Council of Europe. 2019. Available from: https://rm.coe.int/unboxing-artificial-intelligence-10-steps-to-protect-human-rights-reco/1680946e64 | Citation tracking | 2019 | Council of Europe Commissioner for Human Rights | Member states of the Council  of Europe | Disclosure, comprehensibility, usability |
|  | 29 | Council of Europe / Committee of Convention 108. Guidelines on Artificial Intelligence and Data Protection. Consultative Committee of the Convention for the Protection of Individuals with regard to Automatic Processing of Personal Data (Convention 108). 2019. Available from: https://rm.coe.int/guidelines-on-artificial-intelligence-and-data-protection/168091f9d8 | Citation tracking | 2019 | Council of Europe | Governments, AI developers,  manufacturers, service providers | Data basis, reliability, disclosure |
|  | 30 | Council of Europe Committee of Ministers. Recommendation CM/Rec(2019)2 of the Committee of Ministers to member States on the protection of health-related data. 2019. | Citation tracking | 2019 | Council of Europe Committee of Ministers | Member states of the Council  of Europe | Data basis |
|  | 31 | Council of the European Union, Presidency conclusions – The Charter of Fundamental Rights in the context of Artificial Intelligence and Digital Change, 11481/20, October 21, 2020. | Citation tracking | 2020 | Council of the European Union | European Union, member states of the European Union | Data basis, disclosure |
|  | 32 | European Artificial Intelligence Office. AI Pact – Organisations’ commitments. 2024. Available from:  https://digital-strategy.ec.europa.eu/en/policies/ai-pact | Citation tracking | 2024 | European Artificial Intelligence Office | All organisations within the European Union | Disclosure, comprehensibility, usability |
|  | 33 | European Commission, Communication from the Commission to the European Parliament, the European Council, the Council, the European Economic and Social Committee and the Committee of the Regions: Artificial Intelligence for Europe. COM(2018) 237 final, Brussels, 25.4.2018. | Citation tracking | 2018 | European Commission | Member states of the European Union | Usability |
|  | 34 | European Commission. Communication from the Commission to the European Parliament, the Council, the European Economic and Social Committee and the Committee of the Regions: Building Trust in Human-Centric Artificial Intelligence. Brussels, 8.4.2019. COM(2019) 168 final. | Citation tracking | 2019 | European Commission | Member states of the European Union | Data basis, reliability, disclosure, comprehensibility, usability |
|  | 35 | European Commission. Communication from the Commission to the European Parliament, the European Council, the Council, the European Economic and Social Committee and the Committee of the Regions - Coordinated Plan on Artificial Intelligence. Brussels, 7.12.2018 COM(2018) 795 final.  European Commission. ANNEX to the Communication from the Commission to the European Parliament, the European Council, the Council, the European Economic and Social Committee and the Committee of the Regions - Coordinated Plan on Artificial Intelligence. Brussels, 7.12.2018 COM(2018) 795 final ANNEX. | Seed document | 2018 | European Commission | Member states of the European Union as a part of the group on digitising European industry and AI, Norway, Switzerland, Commission of the European Union | Disclosure |
|  | 36 | European Commission. Communication from the Commission to the European Parliament, the Council, the European Economic and Social Committee and the Committee of the Regions -Fostering a European approach to Artificial Intelligence. Brussels, 21.4.2021 COM(2021) 205 final. | Seed document | 2021 | European Commission | Member states of the European Union | Data basis, usability |
|  | 37 | European Commission: Directorate-General for Communications Networks, Content and Technology, High-Level Expert Group on Artificial Intelligence, Ethics guidelines for trustworthy AI. Publications Office; 2019. Available from: doi/10.2759/346720 | Citation tracking | 2019 | European Commission | Europe and beyond: all AI stakeholders | Reliability, disclosure, comprehensibility, usability |
|  | 38 | European Commission for the Efficiency of Justice (CEPEJ) / Council of Europe. European Ethical Charter on the Use of Artificial Intelligence in Judicial Systems and their environment.2018. Available from: https://rm.coe.int/ethical-charter-en-for-publication-4-december-2018/16808f699c | Citation tracking | 2018 | European Commission | Member states of the Council  of Europe | Disclosure |
|  | 39 | European Commission, Secretariat-General. Joint Communication to the European Parliament and the Council - Resilience, Deterrence and Defence: Building strong cybersecurity for the EU. JOIN/2017/0450 final/2. | Citation tracking | 2017 | European Commission, Secretariat-General | European Union, member states of the European Union | Usability |
|  | 40 | European Commission. White Paper on Artificial Intelligence: A European approach to excellence and trust (COM(2020) 65 final). Brussels: European Commission. Available from: https://ec.europa.eu/commission/presscorner/detail/en/ip_20_273 | Citation tracking | 2020 | European Commission | Member states of the European Union, European institutions, all stakeholders | Data basis, reliability, disclosure |
|  | 41 | European Parliament. Civil liability regime for artificial intelligence, European Parliament resolution of 20 October 2020 with recommendations to the Commission on a civil liability  regime for artificial intelligence (2020/2014(INL)) (2021/C 404/05). | Citation tracking | 2020 | European Parliament | European Union | Comprehensibility |
|  | 42 | European Parliament. European Parliament resolution of 20 October 2020 with recommendations to the Commission on a framework of ethical aspects of artificial intelligence, robotics and related technologies (2020/2012(INL)). | Citation tracking | 2020 | European Parliament | European Union, member states of the European Union | Data basis, reliability, disclosure, comprehensibility, usability |
